# A Wireless and Batteryless Intelligent Carbon Monoxide Sensor

**DOI:** 10.3390/s16101568

**Published:** 2016-09-23

**Authors:** Chen-Chia Chen, Gang-Neng Sung, Wen-Ching Chen, Chih-Ting Kuo, Jin-Ju Chue, Chieh-Ming Wu, Chun-Ming Huang

**Affiliations:** National Chip Implementation Center, National Applied Research Laboratories, 7F, No. 26, Prosperity Rd. I, Science Park, Hsinchu City 30078, Taiwan; gnsung@narlabs.org.tw (G.-N.S.); wenching@cic.narl.org.tw (W.-C.C.); ctkuo@narlabs.org.tw (C.-T.K.); jrchu@narlabs.org.tw (J.-J.C.); 0304070@narlabs.org.tw (C.-M. W.); cmhuang@cic.narl.org.tw (C.-M.H.)

**Keywords:** Internet of Things, wireless sensor network, batteryless, carbon monoxide, sensor, embedded system

## Abstract

Carbon monoxide (CO) poisoning from natural gas water heaters is a common household accident in Taiwan. We propose a wireless and batteryless intelligent CO sensor for improving the safety of operating natural gas water heaters. A micro-hydropower generator supplies power to a CO sensor without battery (COSWOB) (2.5 W at a flow rate of 4.2 L/min), and the power consumption of the COSWOB is only ~13 mW. The COSWOB monitors the CO concentration in ambient conditions around natural gas water heaters and transmits it to an intelligent gateway. When the CO level reaches a dangerous level, the COSWOB alarm sounds loudly. Meanwhile, the intelligent gateway also sends a trigger to activate Wi-Fi alarms and sends notifications to the mobile device through the Internet. Our strategy can warn people indoors and outdoors, thereby reducing CO poisoning accidents. We also believe that our technique not only can be used for home security but also can be used in industrial applications (for example, to monitor leak occurrence in a pipeline).

## 1. Introduction

As billions of smart sensors connect to the Internet, one popular Internet of Things (IoT) application features an intelligent home gateway that becomes a central point of connection for smart sensors that monitor the home environment [1,2,3,4,5,6,7,8]. Carbon monoxide (CO) poisoning is a significant cause of illness and death in the United States (US). The internal combustion engine and stoves burning fossil fuels result in most CO poisoning [9]. In contrast to the US, most CO poisoning in Taiwan results from the misuse of gas-powered water heaters [10]. A CO alarm is commonly used for detecting the presence of CO gas in ambient conditions to prevent CO poisoning. A typical CO alarm sounds a warning before the CO level reaches a dangerous level, but it only alerts people nearby. A CO alarm with Wi-Fi function that connects to the Internet to warn people is very rare [11,12,13,14]. Nevertheless, the Wi-Fi communication protocol consumes much energy, which results in lower battery life and battery lifespan as compared with that of a typical stand-alone CO alarm. When a low battery level is achieved, the CO alarm might disconnect from the Internet. In order to overcome a battery reaching a low battery level, a rechargeable battery is used to power the wireless CO alarm. To the best of our knowledge, most indoor CO alarms are powered by a long-lifetime (over a few years) sealed battery. In fact, the CO alarm could possibly fail to function due to battery failure. Taiwan sees especially brutal heat and high humidity in the summer. CO alarms installed in the balcony are easily exposed to high temperature and high humidity conditions, reducing the life of the batteries.

In the past decade, some energy harvesting technologies have already been used to power a wireless sensor node. If we can harvest enough power for an applied wireless sensor, we do not need to worry about sensor failure from running out of battery power. For example, some radio frequency identification (RFID) sensors were driven by using energy harvesting from the RF field [15,16]. A passive RFID sensor does not contain a battery, but most of the low-frequency and high-frequency passive RFIDs provide short read ranges from 10 cm to 1 m. Ultra-high-frequency (UHF) passive RFIDs with a long read range can be as long as 12 m; nevertheless, a UHF RFID cannot be used without a license in most of the world. The best-known energy-harvesting collectors are solar cells and wind turbines, which have become major renewable energy sources. Solar cells and wind turbines are also used to power wireless remote sensors [17,18]. Unfortunately, utilizing solar cells and wind turbines in an indoor environment faces challenges such as low indoor lighting and a lack of wind source. However, a novel self-powered water consumption sensor has been demonstrated [19], and its input energy is only harvested from the flow of water through the micro turbine. 

Ultimately, a batteryless wireless sensor will possibly be a final candidate in the future; it does not adversely impact the environment. To enable batteryless wireless sensors to operate reliably, a power management circuit that monitors energy harvesters and optimizes the use of harvest energy is a key technology. Traditionally, the hydroelectric generator is able to supply power to the electronic system when the flow in the pipe is stable. Otherwise, the energy runs out when the flow cannot push the turbine forward to the hydroelectric generator or when the output energy of the hydroelectric generator is not sufficient to supply the electronic system. However, in many reality conditions, such as in slow or variation flow, the output voltage of the hydroelectric generator is very weak or non-stable. Weak energy cannot enable the system, and non-stable voltage might damage the system too.

**Figure 1:**
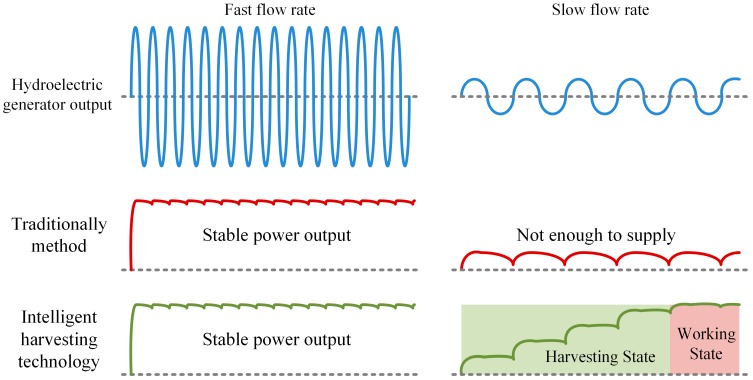
Output power comparison with fast and slow flow rates.

In this study, we use an intelligent hydroelectric energy harvesting technology to power a CO sensor without a battery (COSWOB), whether the flow in the pipe is stable or not. Figure 1 illustrates an output power comparison of the traditional and proposed methods, with fast and slow flow rates.

**Figure 2:**
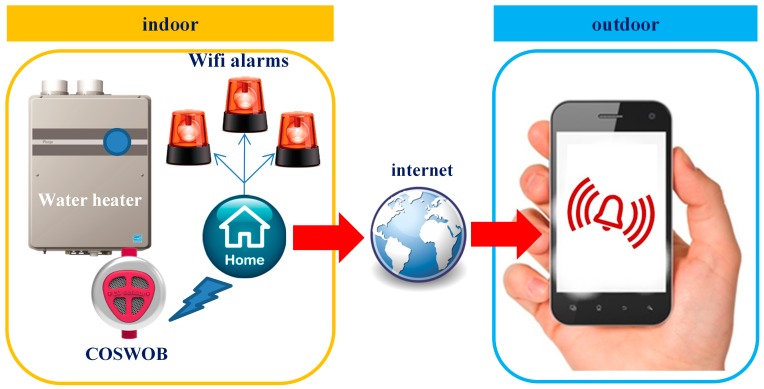
Schematic of the key devices in our strategy for avoiding carbon monoxide (CO) poisoning happen.

Our strategy to prevent CO poisoning by monitoring the CO level by a COSWOB is shown in Figure 2. The COSWOB is installed in a cold water inlet of a natural gas water heater, which is located on a balcony, and the Wi-Fi alarms are placed in another location, such as a bathroom, living room, and bedroom. The COSWOB monitors the CO level in ambient conditions, which is generated from the gas water heater, and transmits these accumulated data to an intelligent gateway through the Bluetooth low-power (BLE) protocol. When the CO level reaches a dangerous level, the COSWOB alarm sounds loudly. Additionally, an application which is run in the intelligent gateway will send a notification to activate Wi-Fi alarms when the COSWOB senses CO over the threshold value in the air. The Wi-Fi alarms can generate louder sounds to alert users in the bathroom and other people in the living room or bedroom. Meanwhile, the intelligent gateway also sends a warning message to smartphones of their family members outdoors in case users miss this louder alarm. 

The typical CO alarm works 24 h per day to detect CO release from the natural gas water heater. However, hot water consumption is mostly concentrated in certain periods in a day, and the natural gas water heater only possibly releases CO during those periods. It is not necessary to detect CO from the natural gas water heater when it is shut down; it will waste battery energy of the typical CO alarm. In contrast to the typical CO alarm, when the natural gas water heater is shut down, the COSWOB is always OFF due to the lack of water flowing through the generator. It is only ON when users need hot water to operate the natural gas water heater. When the cold water flows through the generator of the COSWOB, the COSWOB will turn ON within a few seconds. After the COSWOB is ON, it connects to an intelligent gateway until the COSWOB is OFF. Moreover, typical wireless CO alarms only connect to the Internet when an alarm is activated to extend battery life and battery lifespan. Therefore, the COSWOB can provide early warnings to people before the CO level reaches a dangerous level.

## 2. System Hardware 

**Figure 3:**
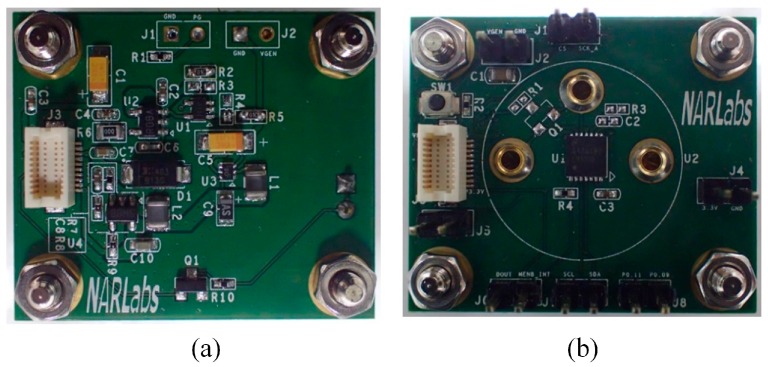

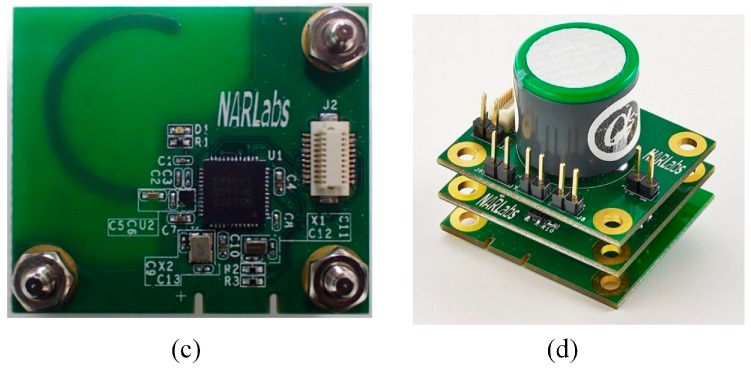
Photographs of the printed circuit board of (**a**) the power module; (**b**) the sensor module; (**c**) the microcontroller module; and (**d**) the complete assembled stack.

The COSWOB consists of three modules—a power module, a sensor module, and a microcontroller module, as shown in Figure 3. Each module has a dimension of 25 mm × 25 mm (width × length), and they are connected through socket connectors with a common power and communication bus.

### 2.1. Power Module

**Figure 4:**
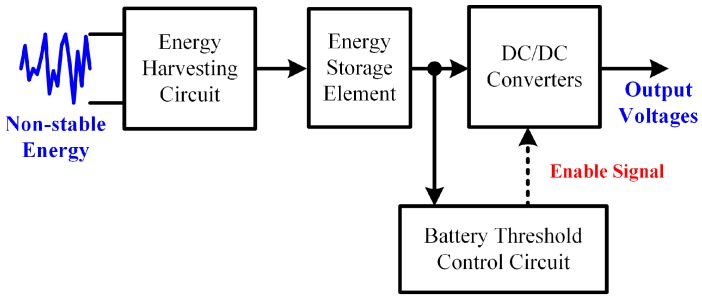
The functional block diagram of power module.

The power module (Figure 3a) is used to harvest energy from the water flow in a pipe of the gas water heater and supply multiple outputs (2.5 V, 3.3 V, and 5 V) to the COSWOB. The power module is composed of three core units: (1) Energy Harvesting with Boost Charger; (2) Voltage Reference Generator; and (3) Step-Up Converter. To drive a sensor readout circuit, 5 V of power supply and 2.5 V of reference voltage are required, and the functional diagram of the power module is shown in Figure 4. In the harvesting state, the Energy Harvesting Circuit collects the non-stable energy from the hydroelectric generator and stores the energy in the Energy Storage Element, which is the 0.1-F supercapacitor in this work. The Battery Threshold Control Circuit monitors the voltage of the supercapacitor. When the voltage of the supercapacitor is greater than 4.5 V, the enable signal is set, and the DC/DC Converters start to work (Working state). On the other hand, the voltage of the supercapacitor starts to drop when the input energy is not enough to supply the sensing system. Until the voltage drops to under 2.3 V, the enable signal is unset and the system goes back to harvesting state. A DC/DC Step-Up Converter and a low-dropout Voltage Reference Generator are proposed in this design. A low-side switch regulator ideal for boost and SEPIC DC/DC regulation is intended to achieve the proposed Step-Up Converter circuit. It provides all the active functions to provide local DC/DC conversion with fast transient response and accurate regulation. Switching frequency is internally set to 1.6 MHz, allowing the use of a tiny surface mount inductor and chip capacitors, while providing efficiencies near 90%. DC/DC regulator used current-mode control and internal compensation methods provides a minimum component count and high-performance regulation over a wide range of operating conditions. The energy harvesting with a boost charger is designed with the flexibility to support a variety of energy storage elements, such as a rechargeable battery, a supercapacitor, or a conventional capacitor. Therefore, a Battery Threshold Control circuit is designed in the sub-circuit. The availability of the sources from which harvesters extract their energy can often be sporadic or time-varying. The energy harvesting with a boost charger will typically need some energy storage element, such as a supercapacitor in the proposed system. The supercapacitor provides constant power to the COSWOB and also allows it to handle any peak currents that cannot directly come from the input source.

### 2.2. Sensor Module

The sensor module (Figure 3b) supports both types of electrochemical sensors (three leads and two leads gas sensors), but three-lead electrochemical CO sensor (CO-AF, Alphasense, Essex, UK) is only used in this study. A potentiostat is required for maintaining appropriate bias potential between the reference and the counter electrodes of the electrochemical gas sensor. We adapt a programmable analog front end (AFE), LMP91000 (Texas Instruments, Dallas, TX, USA), to provide a bias between reference and counter electrodes and convert into a voltage from the output current of work electrodes. An analog-to-digital converter (ADC161S626, Texa Instruments, Dallas, TX, USA) samples the output voltage of the LMP91000 and converts this analog voltage to a digital value for further data gathering by a microcontroller.

### 2.3. Microcontroller Module

The microcontroller (nRF51822, Nordic Semiconductor, Skøyen, Norway) used here is from Nordic, which is built around a 32-bit ARM Cortex^TM^ M0 CPU (Central Processing Unit) with an embedded 2.4-GHz transceiver which supports the BLE protocol. The nRF51822 configures the LMP91000 through Inter-Integrated Circuit (I^2^C) interface and reads the output voltage of the LMP91000 from the ADC161S626 through a Serial Peripheral Interface (SPI) interface. The COSWOB will transmit collected data to the intelligent gateway through the BLE protocol. When the COSWOB is activated, a series of sequences initializes itself. These sequences initialize not only the frequently used peripheral devices such as GPIOs, timers, I2C, and SPI, but also the configurations of the LMP91000 and the BLE services. After initialization, the nRF51822 periodically reads the output voltage of LMP91000 by the ADC data value from the ADC161S626 through the I2C bus. A timer is set to notice the nRF51822 by a specific interrupt every 20 ms. When an interrupt happens, the accumulation of data is operated to calculate the mean (64 sensor data), and the averaged result is sent to the intelligent gateway through the BLE protocol. 

**Figure 5:**
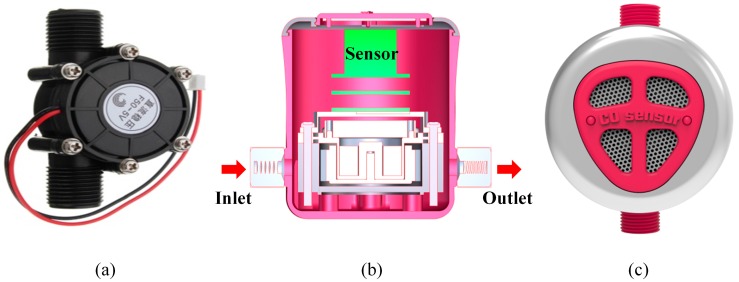
(**a**) A micro-hydropower generator; and (**b**) cross-sectional view and (**c**) top view of the CO sensor without battery (COSWOB).

A micro-hydropower generator (F50-5V) is coupled to the turbine and used to convert the mechanical energy from the water into electrical energy, as shown in Figure 5a. The micro-hydropower generator produces the electric power from the movement of tap water in the pipe of the natural gas water heater, and the output power directly supplies the power module. The maximum output voltage and current are 5 V and >100 mA, respectively. The water pressure of the water outlet closed and open at the maximum voltage are 0.6 Mpa and 1.2 Mpa, respectively. The micro-hydropower generator has a dimension of 88 mm × 58 mm × 39 mm (length × width × height), and its input and output connect thread gauge are G ½’’. We could easily install the COSWOB on a natural gas water heater by using a standard flexible pipe. The complete assembled stack was fixed by screws on the top surface of the micro-hydropower, and the electrochemical CO sensor was then directly mounted on the socket of the PCB (Printed Circuit Board) of the sensor module.

## 3. System Software

An application is run in the intelligent gateway (SZ87R6, Shuttle) for collecting and storing CO sensor data. A Nordic nRF51 Bluetooth dongle is plugged into the USB port of the intelligent gateway. Once the application is launched, it starts to discover numbers of the Bluetooth dongles on it and then generates corresponding control daemons for detecting Bluetooth dongles. The control daemons initialize the corresponding Bluetooth dongles; at the same time, the application generates a data pool for saving data, which is received from the activated COSWOB. The status of the COSWOB is updated instantly on the main page of the server application. While receiving data from the activated COSWOB, the application also transmits the CO level detected by the COSWOB to a smartphone through the Wi-Fi protocol. Moreover, the application also sends a notification to trigger the Wi-Fi alarm if necessary.

We also developed a mobile application for monitoring the COSWOB. The mobile application is available on the Android operating system. Users or their family members can monitor the status of the COSWOB in real-time through mobile devices, such as a smartphone and a tablet. The mobile application listens for the data transmitted from the intelligent gateway. For visual displays, we typically use a numerical indicator and a chart. Moreover, when the detected CO level reaches a dangerous level, the mobile application will alert the user with siren sounds, and a warning sign and the occurrence time will be displayed on the screen to notify users. The warning sign lasts until users dismiss it.

## 4. Calibration Method of the COSWOB

**Figure 6:**
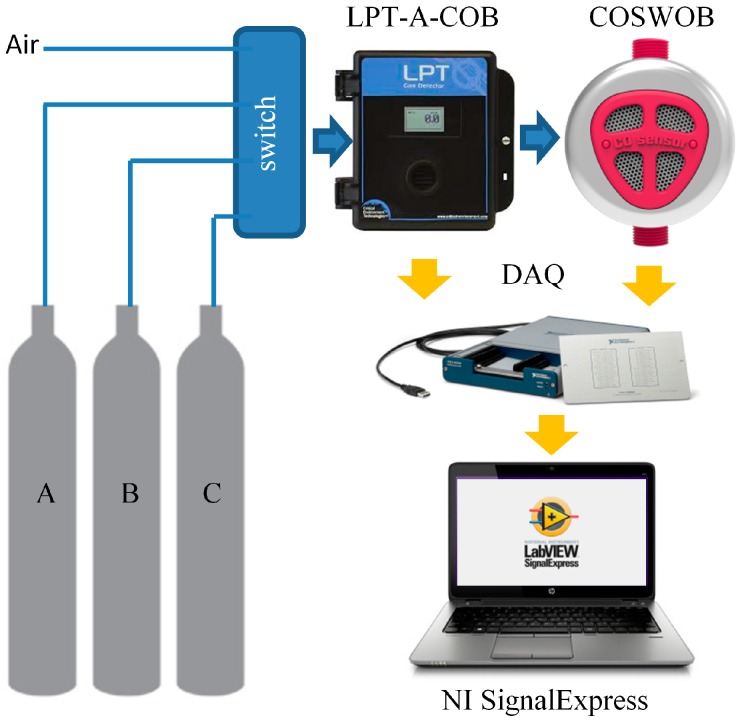
Schematic diagram of a gas sensor calibration system for the COSWOB.

The COSWOB is calibrated and tested using a homemade gas sensor calibration system where known concentrations of CO gas are introduced from the cylinders, as shown in Figure 6. This method works by introducing gas of known concentrations (25, 50, 100 ppm) then performing a check with a gas reference sensor which can be used to calibrate the COSWOB. In order to calibrate the COSWOB, its output signals were also compared to the output signals of a reference sensor (LPT-A-COB, Critical Environment Technologies Canada Inc., Delta, BC, Canada), which provides a high level of accuracy for the measurement of 0–100 ppm CO with 4–20 mA of output. A precision 150-ohm resistor with a 0.1% tolerance is connected to the output terminal of the LPT-A-COB reference sensor. The voltage drop across the precision resistor is measured by data acquisition (DAQ) (USB-6281, National Instrument, Austin, TX, USA), and NI SingnalExpress is used to analyze acquisition data.

## 5. Results and Discussion

**Figure 7:**
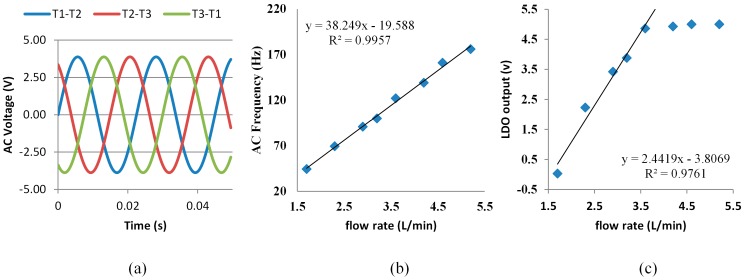
(**a**) The output signals of three loops of the stator at a flow rate of 1.7 L/min; (**b**) Frequency of the sinusoidal waveform of the one of three loops of the stator as a function of the water flow rate; (**c**) The output voltage of the LDO regulator as a function of the water flow rate.

The micro-hydropower generator consists of a rotor with a circular permanent magnet, a stator, and a three-phase AC-DC converter. The stator used the delta connection method to connect windings. The rotor is placed in a flow chamber of the micro-hydropower generator, and blades of the rotor are perpendicular to the flow direction of the tap water. Blades on the rotor are angled to transform energy from the tap flow stream into rotational energy. The micro-hydropower generator is designed to operate in the COSWOB that flows in only one direction. Attempting operation in the reverse direction does not damage the micro-hydropower or other components of the COSWOB. No output voltage of the micro-hydropower generator was observed as an operation in the opposite direction because the rotation efficiency of angled blades in the reverse direction is very small. In order to add the maximum energy to the tap water, the inlet and outlet sizes of the flow chamber are different to improve rotor rotation efficiency. Moreover, the O-ring between the cap and flow chamber keep water from leaking out form the flow chamber of the micro-hydropower generator. A circular permanent magnet is adhered to the inside of the rotor blade. The circular permanent magnet will also move, producing a rotating magnetic field as water flow revolves around the rotor. A sinusoidal waveform is observed between any two winding terminals of the stator, as shown in Figure 7a, and the phase of the output waveform from the stator are equally divided into 120°. The frequency of the sinusoidal waveform as a function of the water flow rate, measured in the flow rate, is in the range 1.7–5.2 L/min, as shown in Figure 7b. Clearly, a linear dependence of the frequency of the sinusoidal waveform on the water flow rate is observed. The three-phase full-wave rectifier consists of a three-phase diode bridge, comprising six diodes. It converts a three-phase AC voltage from the stator to the DC voltage that is periodic over one-sixth of the input AC voltage cycle. To get ripple-free DC voltage, it is regulated to a ripple-free DC voltage using an LDO linear regulator. The output voltage of the LDO regulator as a function of the water flow rate is shown in Figure 7c. The output voltage of LDO regulator reaches a saturation value of 5 V at 4.2 L/min, and its output power is ~2.5 W. Its output voltage becomes tiny under a flow rate of 1.7 L/min. We also test flow rate of a few domestic water pipelines by a flow switch meter (LFE1A3F1, SMC, Tokyo, Japan), which ranges from 3.3 L/min to 8.2 L/min. This means that normal domestic water can directly drive the micro-hydropower generator to power the COSWOB. Additionally, we can also measure the water flow rate by monitoring the frequency of the sinusoidal waveform of one loop of the stator or output voltage of the LDO regulator. Nevertheless, the linear range of the relationship between output voltage of the LDO regulator and the flow rate is from 1.7 L/min to 3.6 L/min. The linear range of the relationship between the frequency of the sinusoidal waveform of any loop of the stator and the flow rate is from 1.7 L/min to 5.3 L/min. This means that we can estimate the water usage based on the monitoring frequency of the sinusoidal waveform of any loop of the stator. 

The flow rate in a real domestic water supply system is not constant. The variable flow rate results in unstable voltage generated by the micro-hydropower generator. It will degrade the performance of the COSWOB. Therefore, we designed a novel power module to overcome this issue. At first, we evaluated that the power consumption of the COSWOB, which includes a three-lead electrochemical CO sensor, a readout circuit, and a microcontroller with BLE, was around 12 mW in total. In our power module, the capacitance of a supercapacitor in the energy harvesting with a boost charger can dominate the operating time of the COSWOB when the energy harvesting from the micro-hydropower generator is tiny; even domestic water is off in the short duration time. We used a 0.1-F supercapacitor as energy storage to store the unstable input energy from the micro-hydropower generator. Five volts is the limit and meant to remain stored in the supercapacitor in the COSWOB. Therefore, the maximum ideal energy stored in the supercomputer is 1.25 J, based on Equation (1). Therefore, 1.25 J equals to 1.25 W/s. When the input power is zero, the energy of the 0.1-F supercapacitor can offer the COSWOB an operating time of at least 1.5 min.

(1) $$E_{\mathrm{stored}}=\frac{1}{2}{\mathrm{CV}}^{2}=0.5\times 0.1\times 5^{2}=1.25\;J.$$

**Figure 8:**
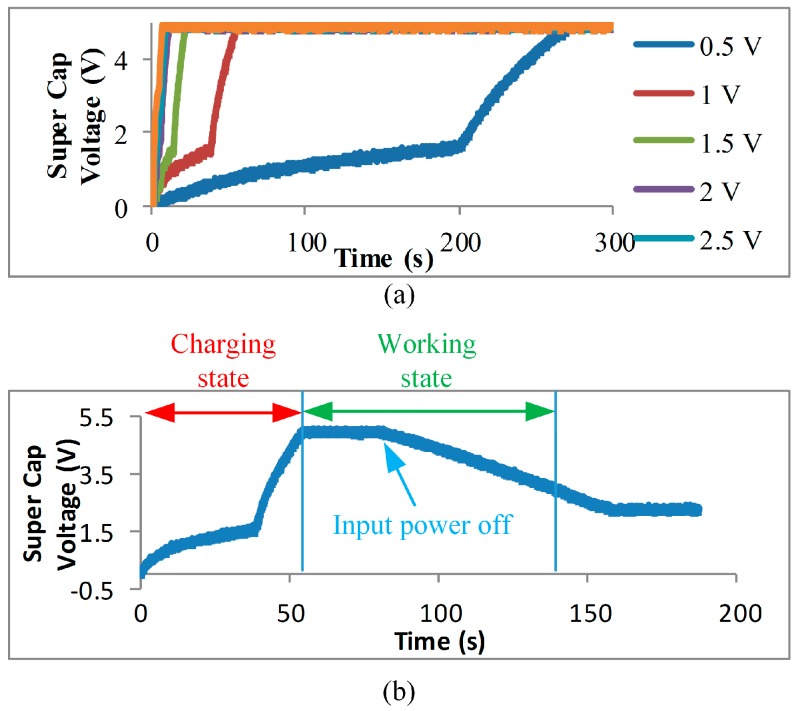
(**a**) The charge curve of sensing system with different input voltages; (**b**) The sensing system operation process.

The intelligent hydroelectric energy harvesting technology in this work composes an energy harvesting circuit, an energy storage element, a battery threshold control circuit, and DC/DC converters. The input can be any non-stable voltage but should be greater than 330 mV. A chart of charges of the supercapacitor with different input voltages is shown in Figure 8a. We experimented with six different input voltages: 3 V, 2.5 V, 2 V, 1.5 V, 1 V, and 0.5 V. The charging times of the output voltage that can reach 5 V are around 7 s, 9 s, 12 s, 23 s, 56 s, and 275 s, respectively. The charge curve has two slopes of charging rate because the energy harvesting circuit has two kinds of charging methods: a trickle current charge and a main boost charge. Morever, the output voltage of micro-hydropower generator is around 1 V (flow rate = 1.9 L/min) and the voltage of the energy storage element can be charge to almost 4.6 V. In this condition, a microcontroller module (wireless communication function) and sensor module (sensor function) cannot work under a supply voltage of around 1 V when we only used the micro-hydropower generator to supply the COSWOB without a power module. Figure 8 shows the sensing system operation process with 1 V of charging and discharging. The sensing system turns to a working state when the voltage of the supercapacitor is greater than 4.5 V, and it turns off when the voltage of the supercapacitor is lower than 2.3 V. In this work, we use the 0.1-F supercapacitor that can still work around 75 s when the hydroelectric generator is turned off.

**Figure 9:**
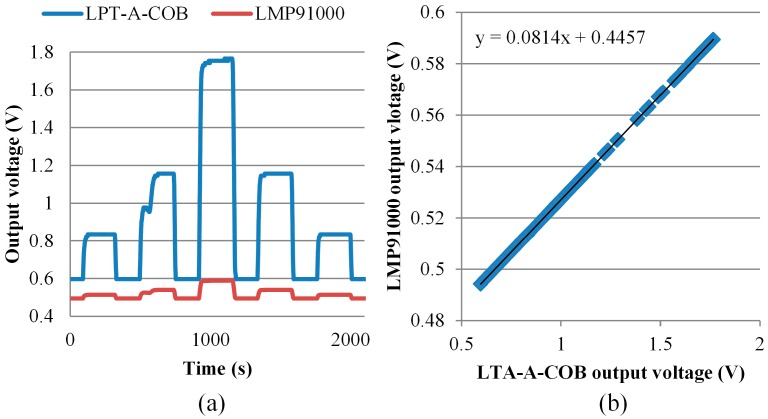
(**a**) Dynamic response curves of the output voltage of the COSWOB and the LPT-A-COB versus time for CO concentrations ranging from 25 to 1000 ppm; (**b**) Linear regression of the relationship between the COSWOB and the LPT-A-COB.

For a calibration of the COSWOB, it is compared with a gas reference sensor. The output voltage of the COSWOB and the LPT-A-COB reference gas sensor upon exposure to CO gas was monitored and recorded as the output voltage. In Figure 9a, the dynamic output voltage versus time for both sensors is demonstrated, with the CO concentration varying from 25 to 100 ppm. No signification hysteresis was observed in both sensors. We used linear regression to estimate the parameters of that relationship between the COSWOB and the LPT-A-COB and obtained formulas for linear regression. The R-squared value (R^2^ = 0.9973) gets closer to one, as shown in Figure 9b; it indicates that a perfect linear relationship between the COSWOB and the LPT-A-COB. We know the sensitivity of the LPT-A-COB is ~11.9 mV/ppm. After getting the relationship between the two sensors, we can calibrate the COSWOB. The first, a bias of the COSWOB, was performed by introducing free air; the bias was ~0.509 V. A zero calibration was then made. After calculation, the sensitivity of the COSWOB was ~1.03 ppm/mV. After calibrating, Equation (2) can be used to calculate the CO level in ambient conditions:

(2) $${\mathrm{CO}}_{\mathrm{Level}}\;\left(\mathrm{ppm}\right)=1.03\times V_{\mathrm{COSWOB}}-509,$$

where CO_Level_ is CO concentration in ambient conditions, and V_COSWOB_ is the output voltage of the COSWOB and is in mV.

**Figure 10:**
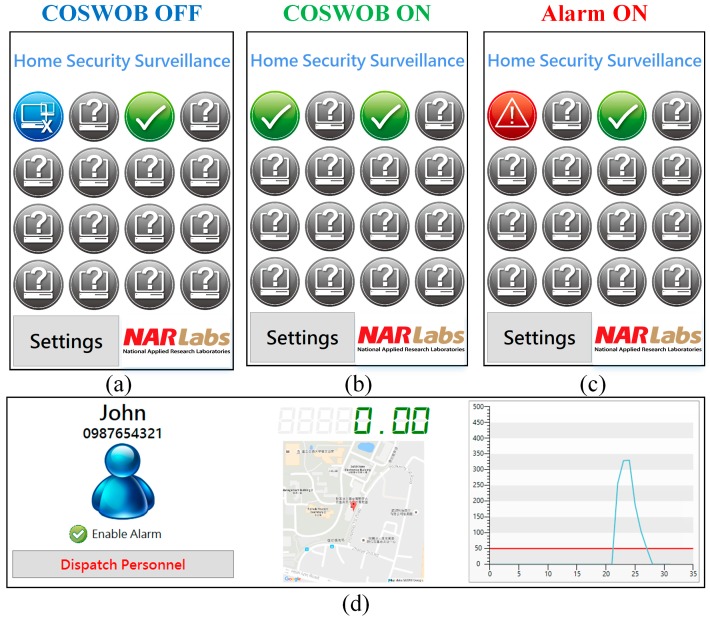
Screenshots of the main pages of the application run in the intelligent gateway, various states: (**a**) COSWOB OFF; (**b**) COSWOB ON; and (**c**) alarm activated; (**d**) Screenshot of the information of the COSWOB.

**Figure 11:**
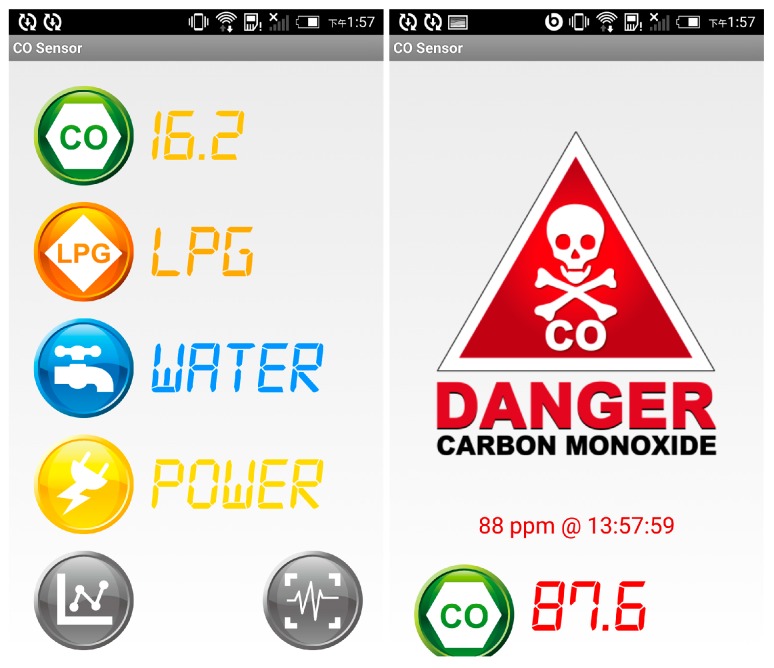
Screenshots of the mobile application for the COSWOB data visualization.

Once the application on the intelligent gateway is launched, the application automatically started to discover COSWOBs nearby. After finding the COSWOBs, its status is updated instantly on the main page of the server application, as shown in Figure 10a–c. If the COSWOB is OFF, the indicator icon on the main page of the application displays OFF status, as shown at row = 1 and column = 1 in Figure 10a. The application repeats to scan any wake-up COSWOB every 30 s (not optimal) until the activated COSWOB is connected to the intelligent gateway. After the COSWOB is connected to the intelligent gateway, the indicator icon on the main page quickly changes to the ON status from the OFF status, and the application starts to continuously receive the data from the COSWOB every second. When the CO level exceeds a dangerous level (50 ppm), the indicator icon is quickly changed to the alarm icon, as shown in Figure 10c. Meanwhile, an information page of the COSWOB automatically pops up, the CO level around the natural gas water heater is real-time displayed on a chart, and the user can overview the history of the CO level, as shown in Figure 10d. The Wi-Fi alarms (TuneBox, Nexum, Taipei, Taiwan) in the other location will generate a loud sound to alert other people. Additionally, the value of the CO level is also transmitted to smartphones that belong to other family members; the mobile application will alert family members to take action when the CO level reaches a dangerous level, as shown in Figure 11. 

## 6. Conclusions

We have demonstrated that our application will be a great benefit for people to avoid CO poisoning from gas water heaters. Our application not only alerts users and their family members inside the house, but also notifies family members outside the house, when CO levels reach a dangerous level. Moreover, our COSWOB is only powered by the micro-hydropower generator. There is no danger that people might be living with a dangerous CO level, or that the COSWOB might not work due to a loss of battery life. The domestic liquefied petroleum gas leakage is also an important issue; the sensor module of the COSWOB can easily integrate with three-lead electrochemical natural gas (NG) or LPG sensor. The next step will be to monitor CO and LPG at the same time in the next generation of COSWOBs.
